# Does SOFA-2 improve mortality prediction in sepsis? A retrospective single-center observational cohort study comparing SOFA-1 and SOFA-2

**DOI:** 10.1186/s12879-026-13829-y

**Published:** 2026-06-20

**Authors:** Kamil Deveci, Maruf Boran, Kübra Elif Bahar, Fatma Sarıdağ, Esma Eryılmaz Eren

**Affiliations:** 1 Division of Intensive Care Unit, Department of Internal Medicine, Kayseri City Education and Research Hospital, Kayseri, Türkiye; 2 Department of Internal Medicine, Kayseri City Education and Research Hospital, Kayseri, Türkiye; 3 Department of Infectious Diseases and Clinical Microbiology, Kayseri City Education and Research Hospital, Kayseri, Türkiye

**Keywords:** Mortality, SOFA-2, Organ failure, Severity of illness index

## Abstract

**Background:**

The Sequential Organ Failure Assessment (SOFA) score has been widely used for nearly three decades to evaluate organ dysfunction and predict mortality in patients with sepsis. Advances in critical care practice have led to the development of an updated version, SOFA-2, incorporating contemporary organ support strategies and revised clinical thresholds. However, real-world comparative data evaluating the prognostic performance of the original SOFA score (SOFA-1) and SOFA-2 remain limited. This study aimed to compare the prognostic performance of SOFA-1 and SOFA-2 for predicting ICU, 28-day, and 90-day mortality in patients with sepsis.

**Methods:**

This retrospective, single-center observational cohort study included adult patients (≥ 18 years) diagnosed with sepsis according to Sepsis-3 criteria and admitted between December 2023 and August 2024. SOFA-1 and SOFA-2 scores were calculated using clinical and laboratory data obtained at ICU admission. The primary outcome was ICU mortality; secondary outcomes were 28-day and 90-day mortality. Multivariable logistic regression was performed to identify independent predictors of mortality. Discriminatory performance was assessed using receiver operating characteristic analysis, and areas under the curve were compared using DeLong’s test. Optimal cut-off values were determined using the Youden index.

**Results:**

Among 417 screened patients, 222 met the inclusion criteria. ICU mortality was 57.7%. Each one-point increase in SOFA-1 and SOFA-2 scores was associated with a 42% and 43% increase in ICU mortality, respectively (*p* < 0.001 for both). The area under the curve for ICU mortality prediction was 0.843 (95% CI 0.792–0.895) for SOFA-1 and 0.845 (95% CI 0.795–0.896) for SOFA-2; the difference between the two scores was not statistically significant (*p* = 0.79). SOFA-2 demonstrated slightly higher specificity, whereas SOFA-1 showed marginally higher sensitivity at the optimal cut-off value.

**Conclusions:**

SOFA-1 and SOFA-2 demonstrated good discriminatory performance for predicting ICU mortality in this cohort. Although no statistically significant difference was observed between the two scoring systems, larger prospective multicenter studies are needed to determine whether clinically meaningful differences exist and to further evaluate the potential advantages of SOFA-2.

**Trial registration:**

Not applicable.

## Background

Every year, significant transformations are taking place in intensive care medicine. Advances in pharmacological therapies, organ support technologies, and evidence-based protocols have substantially improved the delivery and quality of intensive care. Thirty years have passed since the Sequential Organ Failure Assessment (SOFA) score was first used in 1996 [1], and clinical practice has advanced significantly. Treatments we previously used, such as low-dose dopamine [2], have been replaced in clinical practice with specific vasopressors such as norepinephrine and vasopressin [3, 4]. These changes are not only related to cardiovascular support systems; the use of non-invasive respiratory support systems has also increased in our clinical practice [5].

Despite these developments, the original SOFA score (hereafter referred to as SOFA-1) has remained unchanged. SOFA-1 assesses six organ systems (neurological, respiratory, renal, hepatic, cardiovascular, and coagulation) and generates a score between 0 and 24 to predict disease severity and mortality risk [1]. This scoring system cannot account for current, less invasive pharmacological and device-based organ support strategies.

Given the substantial evolution in critical care management, an updated organ dysfunction scoring system applicable across both high-income and low-to-middle-income settings is warranted [6–8]. In 2025, SOFA-2 was introduced as a revised model incorporating contemporary organ support therapies, modified cut-off values, and refined clinical criteria [9]. Compared with the original SOFA score, SOFA-2 includes several updates, including revised vasopressor thresholds, expanded definitions of respiratory support such as high-flow nasal oxygen and non-invasive ventilation, consideration of sedative medication use in neurological assessment, and updated renal support criteria including modern renal replacement therapies. While preserving the original organ dysfunction framework, SOFA-2 was developed to improve applicability to current ICU practice and evolving organ support strategies. Nevertheless, real-world comparative data evaluating the prognostic performance of SOFA-1 and SOFA-2 in patients with sepsis remain limited.

In this study, we aimed to compare the prognostic accuracy of SOFA-1 and SOFA-2 in predicting ICU, 28 and 90-day mortality in patients with sepsis using our institutional cohort.

## Methods

### Study design and setting

This retrospective, single-center observational study was conducted in the Medical Intensive Care Unit-3 (ICU) of Kayseri City Education and Research Hospital, a tertiary referral center providing advanced care for critically ill medical patients. The study was approved by the Ethics Committee of Kayseri City Education and Research Hospital (Decision No: 711, Date: January 7, 2026). Given the retrospective and anonymized nature of the analysis, written patient consent was waived. All procedures adhered to the Declaration of Helsinki, institutional standards, and applicable guidelines. To enhance transparency, this study was reported following the STROBE (Strengthening the Reporting of Observational Studies in Epidemiology) guidelines [10]. Our primary objective is to determine whether there is a difference in the prediction of in-ICU mortality between SOFA-1 and SOFA-2 scores. Our secondary objective is to investigate whether there is a difference in the prediction of 28 and 90 days mortality.

### Patient selection

**Fig. 1:**
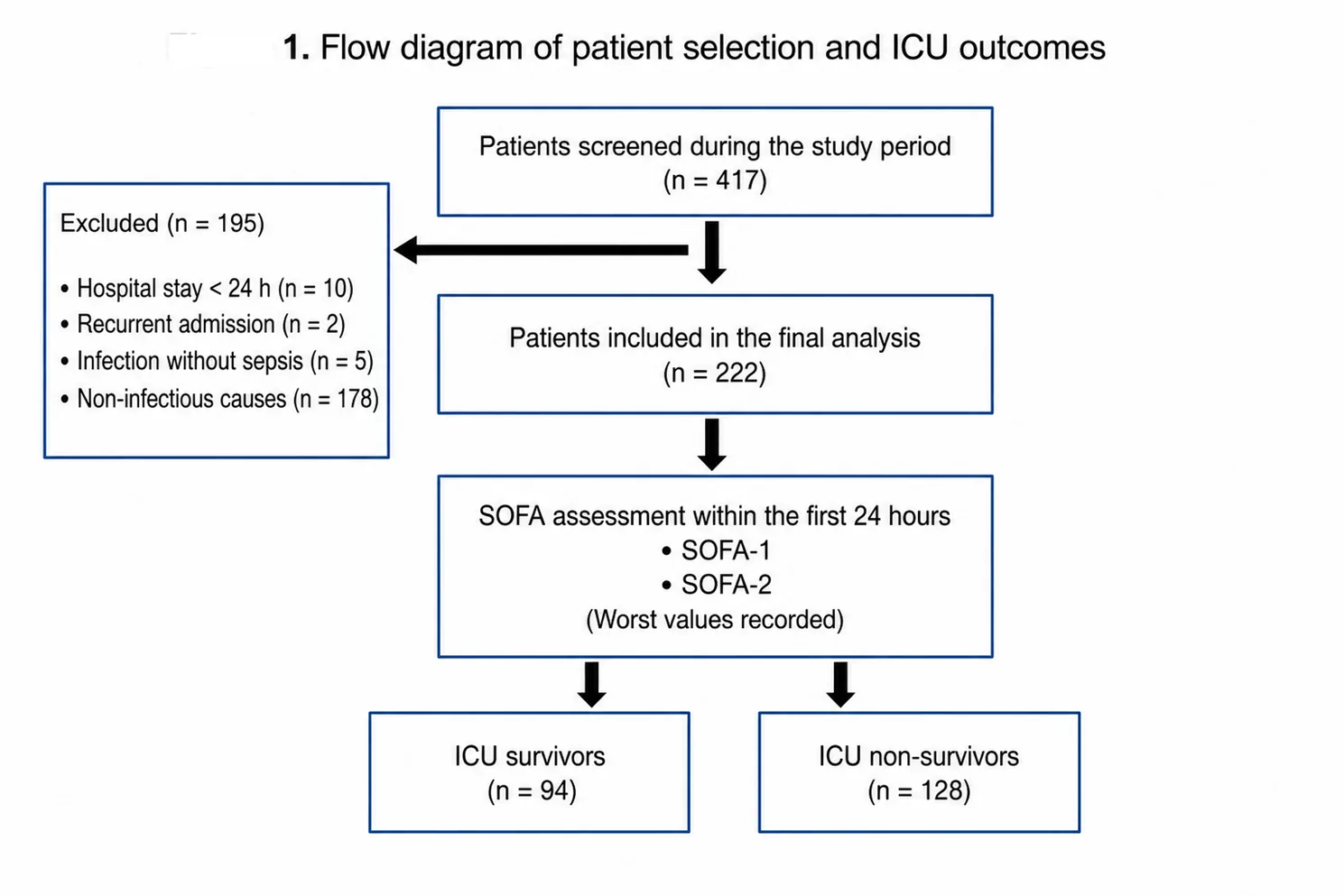
Flow diagram of patient selection and ICU outcomes

The study included all consecutive patients aged 18 years and older diagnosed with sepsis who met the Sepsis-3 criteria [11] and were admitted between December 2023 and August 2024. A total of 417 patients were evaluated, and 222 patients who had all the data necessary to calculate SOFA-1 and SOFA-2 scores and who met the documentation criteria for ICU, 28 and 90 day mortality records were included in the study. Exclusion Criteria: Missing key clinical or laboratory parameters preventing SOFA score calculation. Hospital stay of less than 24 h. Repeat hospitalizations (only the first admission included). Patients lower 18 years of age. Patients without a suspected or confirmed focus of infection. Patients with infection but a sofa score < 2. The flowchart is presented in Fig. 1.

### Data collection

Retrospective review of the hospital electronic health record system (KEYDATA) was performed to extract patient information, encompassing demographic, clinical, treatment and laboratory parameters. For all patients, the following data were recorded: Demographic variables: age, sex, ICU admission area (Ward, Emergency Department, other ICU) and comorbidities. Clinical and treatment variables: GCS score, APACHE-II score, Initial vital signs, vasopressor use, oxygen/ventilation methods (mechanical ventilation, non-invasive ventilation, any advanced ventilator support), need for renal replacement (intermittent or continuous), urine output, source of infection, and antibiotic use were recorded. Laboratory parameters: complete blood cell (CBC) analysis, PaO₂/FiO₂ ratio, biochemical markers (blood urea nitrogen, creatinine, AST, ALT, total bilirubin), inflammatory markers (C-reactive protein (CRP), procalcitonin (PCT)), and lactate level. SOFA-1 and SOFA-2 scores were calculated retrospectively for all patients using clinical, treatment, and laboratory variables at the time of admission to the ICU.

### Disclosure of AI usage

During the preparation of this manuscript, the authors used AI tools ChatGPT by OpenAI (version ChatGPT-5 Thinking/Pro) to improve language and readability. All outputs were reviewed, adapted and integrated into the final work by the authors, who take full responsibility for all content presented herein.

### Definitions

Vasopressor Requirement: 0.05 mcg/kg/min and equivalent norepinephrine requirement was stated. Non-infectious patients: Clinical, laboratory, or radiological findings do not indicate infection, and the current clinical symptoms can be attributed to a non-infectious condition (e.g., acute kidney injury, gastrointestinal bleeding, electrolyte imbalance, vertigo, cerebrovascular disease, hypercalcemia, congestive heart failure, acute coronary syndrome, acute pancreatitis, malignancy-related processes, status epilepticus, pulmonary embolism, etc.), they were defined as non-infectious. Infection patients without sepsis: defined as the absence of signs of organ dysfunction (SOFA < 2) in addition to a clinically consistent focus of infection (pneumonia, urinary tract infection, intra-abdominal infection, skin/soft tissue infection, catheter infection, cholecystitis, central nervous system infection, etc.) supported by physical examination, laboratory findings (including microbiological), and/or imaging studies. Sepsis is diagnosed according to Sepsis-3 criteria. Patients showing signs of infection-related organ dysfunction (SOFA ≥ 2) were classified in this group. SOFA-1: The original scoring system described by Singer et al. [11], evaluating six organ systems using traditional thresholds and clinical measures. SOFA-2: A revised scoring model incorporating updated cut-off values, expanded definitions of respiratory and renal support, and contemporary clinical practices [9]. Mortality Rate: All-cause mortality rates during intensive care unit admission or within 28 and 90 days. All diagnoses were determined retrospectively based on electronic medical records.

Ethical approval was obtained from the local ethics committee prior to the study.

### Statistical analysis

Statistical analyses were conducted using IBM SPSS Statistics version 27.0. Data distribution was evaluated visually and with the Shapiro-Wilk test. Continuous variables were summarized as mean±standard deviation or median (IQR, 25–75%), as appropriate. Continuous variables were compared using the Student’s t-test or Mann–Whitney U test, as appropriate. Categorical variables were analyzed using Pearson’s chi-square or exact chi-square tests. Multivariable logistic regression was applied to determine independent predictors of mortality. Variables were selected a priori according to clinical relevance and univariate associations. All selected variables were simultaneously entered into the multivariable logistic regression model using the ENTER method. Results are presented as odds ratios (ORs) with 95% confidence intervals (CIs). To reduce collinearity and enhance interpretability, composite severity scores (APACHE II, SOFA-1, and SOFA-2) and their individual components were evaluated in separate models. SOFA-1 and SOFA-2 were evaluated for their ability to predict ICU, 28 and 90 days mortality using ROC curve analysis, with AUCs and 95% CIs reported. Correlated AUCs were compared with DeLong’s test (MedCalc). Cutoff points were determined by the Youden index, and diagnostic performance metrics (sensitivity, specificity, PPV, NPV) were calculated from 2 × 2 tables. Statistical significance was defined as a two-tailed *p* < 0.05.

## Results

### Study population and patient characteristics

A total of 417 patients were screened for our study, and 222 patients meeting the inclusion criteria were enrolled (Fig. 1). Women were 38% (*n* = 85) of our patients and the age of all patients was 70 (62–80), with median SOFA-1 score 9 (5–13), SOFA-2 score 8 [5–12], mean APACHE-II score of 25.8 ± 9.9. Our ICU mortality rate was 57.7% (*n* = 128), indicating a cohort with a high severity of illness. The most common comorbid condition was hypertension (Table 1).

**Table 1:** Baseline characteristics of the study population

Variable	Value
Age years	70 (62–80)
Female Sex, n (%)	85 (38)
ICU admission n (%)	
Emergency Department	125 (56.3)
Ward	79 (35.6)
Other ICU	18 (8.1)
Comorbities, n (%)	
Hypertension	97 (18.5)
Solid malignancy	74 (14.1)
Diabetes mellitus	72 (13.8)
Chronic kidney disease	47 (9)
Coronary artery disease	42 (8)
COPD	30 (5.7)
GCS Score	12 (5–14)
APACHE-II Score	25.8 ± 9.9
SOFA-1 Score	9 (5–13)
SOFA-2 Score	8 (5–12)
ICU mortality n (%)	128 (57.7)
28 days mortality n (%)	120 (54)
90 days mortality n (%)	143 (64)

Abbreviations: APACHE, Acute Physiology and Chronic Health Evaluation; GCS, Glasgow Coma Scale; ICU, Intensive Care Unit; SOFA, Sequential Organ Failure Assessment

### Association of organ dysfunction scores with ICU mortality in critically Ill patients

During the ICU course, patients were classified into survivor (SG) and non-survivor groups (NSG); however, no significant differences were identified between the groups regarding; age, gender, creatinine, pH, PCT, CRP and lymphocyte levels both group. APACHE-II, SOFA-1, and SOFA-2 scores were statistically significantly higher in the non-survivor group (all *p* < 0.001). All variables comprising SOFA, except creatinine, were statistically significantly higher in the NSG group (Table 2).

**Table 2:** Comparison of Survivors and Non-Survivors in the ICU

Variable	Survivors (*n* = 94)	Non-survivors (*n* = 128)	*p*
Age years	71.5 (60–82)	70 (63-79.7)	0.994
Female Sex, n (%)	37 (39.4)	48 (37.5)	0.782
Apache-II Score	**21 ± 7.8**	**29 ± 10.1**	**< 0.001**
SOFA-1 Score	**5 (3–8)**	**12 (8.2–14.7)**	**< 0.001**
SOFA-2 Score	**5 (3–7)**	**11 (7.2–14)**	**< 0.001**
GCS Score	**14 (11-14.2)**	**8 (3–13)**	**< 0.001**
PaO_2/_FiO_2_ ratio	**276 ± 102**	**213 ± 95**	**< 0.001**
Vasopressor requirement n (%)	**20 (21.3)**	**108 (84.4)**	**< 0.001**
**Laboratory variables**			
WBC count*	12.5 (8.6–17.7)	13.1 (7.1–21.5)	0.30
Lymphocyte count *	0.9 (0.55–1.44)	0.81 (0.52–1.47)	0.930
Platelet count *	**195 (144–270)**	**164 (71–264)**	**0.023**
Total bilirubin mg/dL	**0.6 (0.4–1.2)**	**0.8 (0.5–1.97)**	**0.005**
Creatinin mg/dL	1.28 (0.73–3.2)	1.4 (1.2–3.2)	0.144
INR	**1.25 (1.1–1.4)**	**1.4 (1.2–1.87)**	**< 0.001**
pH	7.38 (7.30–7.41)	7.31 (7.24–7.43)	0.104
Lactate mmol/L	**1.5 (1.1–2.5)**	**2.5 (1.4–4.1)**	**< 0.001**
PCT ng/mL	1.3 (0.3–6.4)	1.5 (0.37–6.4)	0.802
CRP mg/L	142 (79–200)	115 (43–208)	0.224

Abbreviations: APACHE-II, Acute Physiology and Chronic Health Evaluation II; GCS, Glasgow Coma Scale; SOFA, Sequential Organ Failure Assessment; WBC, White Blood Cells; *×10^3^/mm^3^

### Univariate and multivariate logistic regression analysis for predictors of ICU mortality

In the multivariate analysis, which included variables that showed meaningful differences between SG and NSG in the univariate analysis, vasopressor requirement (OR 10.8, 95% CI 5.04–23.2; *p* < 0.001) and GCS score (OR 0.82, 95% CI 0.73–0.92; *p* = 0.001) were identified as independent predictors of mortality. Patients requiring vasopressor support had a markedly increased risk of mortality. We found that a one-point decrease in GCS increased ICU mortality by 19%. Overall, the model demonstrated good explanatory performance, accounting for approximately 56% of the variance in mortality (Table 3).

**Table 3:** Univariate and multivariate logistic regression analysis for predictors of ICU mortality

	Univariate OR(%95 CI)	*p* value	Multivariate OR(% 95 CI)	*p* value
GCS Score	**0.817 (0.726–0.919)**	**< 0.001**	**0.817 (0.726–0.919)**	**0.001**
PaO_2_/FiO_2_ ratio	**0.997 (0.993–1.001)**	**< 0.001**	---	0.177
Vasopressor requirement	**10.8 (5.04–23.2)**	**< 0.001**	**10.8 (5.04–23.2)**	**< 0.001**
Platelet count	1.00(1.00–1.00)	0.205	---	0.142
Total Bilirubin mg/dL	1.01 (0.832–1.226)	0.096	---	0.921
INR	1.56 (0.77–3.1)	0.294	---	0.216
Lactate mmol/L	**1.08 (0.892–1.314)**	**0.001**	---	0.423

Abbreviations: CI, Confidence Interval; GCS, Glasgow Coma Scale; OR, Odds ratio

Model fit: −2 Log likelihood = 173; Cox & Snell R² = 0.42; Nagelkerke R² = 0.567

### Each one-point increase in SOFA-1 and SOFA-2 confers a similar rise in ICU mortality

We found that each point rise in the SOFA-1 score was associated with a 42% increase in ICU mortality, while an increase in the SOFA-2 score was associated with a 43% increase (OR 1.428, 95% CI 1.3–1.56; *p* < 0.001 vs. OR 1.43, 95% CI 1.3–1.575; *p* < 0.001).

### SOFA-1 and SOFA-2 demonstrate similar AUCs for ICU mortality prediction

**Fig. 2:**
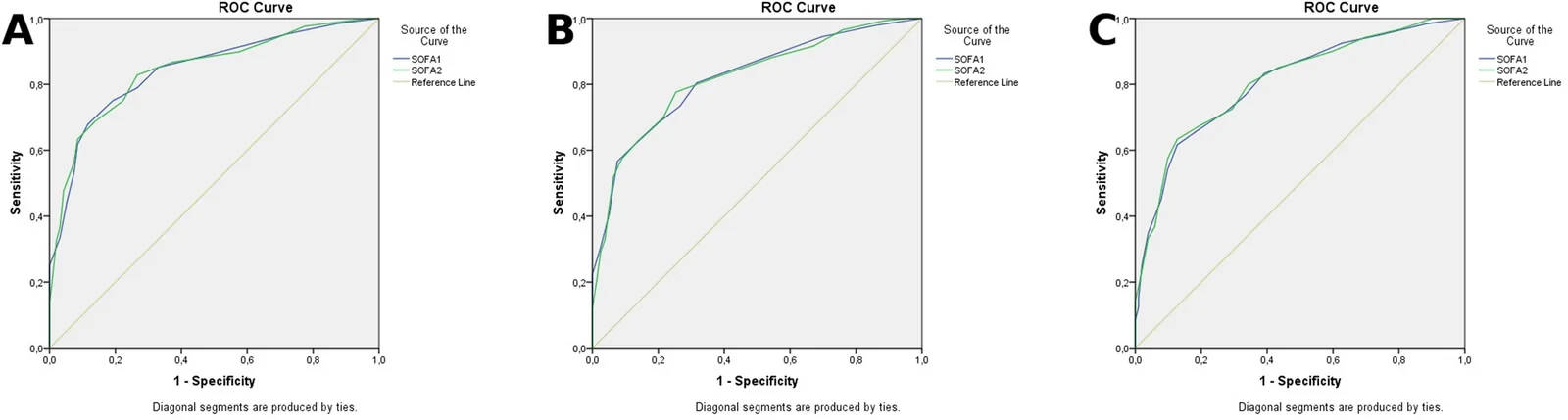
ROC Curves of SOFA-1 and SOFA-2 for ICU(**A**), 28(**B**) and 90(**C**) day Mortality

In a total of 222 patients, ICU mortality occurred in 128 patients (57.7%). The AUC of SOFA-1 for predicting ICU mortality was 0.843 (95% CI: 0.792–0.895), while the AUC of SOFA-2 was 0.845 (95% CI: 0.795–0.896). The difference between the two AUCs was not statistically meaningful (*p* = 0.790). According to the Youden index, an optimal cut-off value of 6 was identified for both scores. At this threshold, diagnostic performance metrics including sensitivity, specificity, PPV, and NPV were calculated. SOFA-2 exhibited higher specificity and PPV, whereas SOFA-1 showed higher sensitivity and NPV at the identified cut-off value (Table 4; Fig. 2).

**Table 4:** Analysis of the performance of SOFA-1 and SOFA-2 in predicting in-ICU, 28 and 90 days mortality in sepsis patients

Outcome	Score	Cut-off	Sensitivity (%)	Specificity (%)	PPV	NPV	AUC	*P*
ICU mortality	SOFA-1	≥ 6	89	51	71.2	77.4	0.843	**< 0.001**
	SOFA-2	≥ 6	86	62.8	71	77.6	0.845	**< 0.001**
28-day mortality	SOFA-1	≥ 6	88.3	47.1	66.3	77.4	0.806	**< 0.001**
	SOFA-2	≥ 6	85.0	56.9	69.9	76	0.810	**< 0.001**
90-day mortality	SOFA-1	≥ 6	86.0	53.2	76.9	67.7	0.819	**< 0.001**
	SOFA-2	≥ 6	81.8	63.3	80	65.8	0.819	**< 0.001**

Abbreviations: AUC, Area under curve; ICU, Intensive care unit; NPV, Negative predictive value; PPV, Positive predictive value

There was no statistically significant difference between SOFA-1 and SOFA-2 AUCs (DeLong test)

## Discussion

The patients included in our study represented a severely ill ICU population with a high burden of organ dysfunction. The median SOFA-1 score for these patients was 9, the median SOFA-2 score was 8 and the mean APACHE-II score was 25.8 ± 9.9. Multivariate analysis identified vasopressor requirement (OR 10.8) and decreased GCS (0.81) as the strongest predictors of mortality. SOFA-1 and SOFA-2 demonstrated good discriminatory performance for predicting ICU mortality, with AUC values of 0.843 and 0.845, respectively (*p* = 0.79). Although no significant difference was observed between SOFA-1 and SOFA-2 in our cohort, this finding may be related to the exceptionally severe nature of the study population, in which advanced sepsis-related organ dysfunction was already present in most patients. Because both scoring systems assess the same fundamental domains of organ dysfunction, they may perform similarly in such high-acuity patients, where severe physiological derangements are already well established. In this context, the ability of SOFA-2 to demonstrate additional discriminatory value may be limited. Nevertheless, SOFA-2 was designed to better reflect contemporary intensive care practice by incorporating updated organ support definitions and thresholds, including high-flow nasal oxygen, non-invasive ventilation, contemporary renal replacement therapies, extracorporeal membrane oxygenation (ECMO), revised vasopressor criteria, and modern approaches to neurological assessment. Therefore, its potential advantages may become more apparent in less severely ill or more heterogeneous ICU populations, where these contemporary support modalities are more likely to influence the assessment of organ dysfunction and risk stratification.

In a multicenter study involving 3.34 million patients, the predicted ICU mortality rate ranged from 12.4% to 31%, whereas the observed mortality rate was 8.1%, varying between 4.5% and 20% across centers. In the same study, the AUC values of SOFA-1 and SOFA-2 for mortality prediction were 0.81 and 0.80, respectively, and a two-stage validation analysis demonstrated no significant difference between the two scoring systems [9]. Compared with that cohort, our study included a substantially higher-risk patient population. Based on the mean APACHE-II score (25.8 ± 9.9), the predicted ICU mortality rate was estimated at 45–60%, while the observed mortality rate was 57%. Although no significant difference was observed between the AUC values in our cohort, improvements in contemporary scoring systems may not necessarily translate into substantially higher AUC values, as discrimination represents only one aspect of prognostic model performance. The potential advantages of SOFA-2 may instead relate to improved calibration and better alignment with current ICU practices and organ support strategies. In the same study, a 1-point increase in the SOFA-2 score was associated with a 37% increase in mortality [9]. In our cohort, each 1-point increase in SOFA-2 and SOFA-1 was associated with a 43% and 42% increase in mortality, respectively. This difference may be attributable to the higher baseline SOFA scores and greater burden of organ dysfunction in our cohort.

Using the Youden index, an optimal cut-off value of ≥ 6 was identified for both scores. For SOFA-1, this threshold yielded a high sensitivity (89%) with moderate specificity (59%), whereas SOFA-2 showed a slightly lower sensitivity (86%) but slightly higher specificity (62.8%), while PPV and NPV values were comparable between the two scores. Previous studies have reported lower discriminatory performance for SOFA-1 when higher cut-off values were applied. For example, a study using a SOFA-1 cut-off value greater than 9 reported an AUC of 0.726 for predicting in-hospital mortality, with a sensitivity of 65% and specificity of 75% [12]. Despite using a lower cut-off value, our study demonstrated a substantially higher AUC, indicating superior overall discrimination. This apparent discrepancy can be explained by differences in study populations, outcome definitions (ICU mortality versus in-hospital mortality), and the methodology used to determine optimal cut-off values.

This study has several limitations. Its single-center, retrospective design and the inclusion of a high-severity sepsis cohort from a tertiary referral medical ICU may limit the generalizability of the findings to other ICU settings. SOFA-1 and SOFA-2 scores were calculated using the worst values within the first 24 h of ICU admission; therefore, dynamic changes in organ dysfunction over time were not evaluated. The study population consisted of a severely ill sepsis cohort with a high ICU mortality rate, which may have influenced the discriminatory performance of both scores and limited the ability to detect small differences between them. Finally, although DeLong’s test was used for AUC comparison, the sample size may not have been sufficient to identify modest differences in predictive performance. Because this study was not designed as a formal equivalence or non-inferiority analysis, the absence of a statistically significant difference between the AUC values should not be interpreted as proof of true equivalence between SOFA-1 and SOFA-2.

## Conclusion

In this study, SOFA-1 and SOFA-2 demonstrated good discriminatory performance for predicting ICU mortality. Although no statistically significant difference in predictive performance was observed between the two scoring systems, this finding should be interpreted cautiously given the relatively limited sample size and the substantial overlap between the scores. The potential advantages of SOFA-2 may lie in its alignment with contemporary organ support practices rather than in improved discrimination within this severely ill cohort. Larger prospective multicenter studies are needed to determine whether clinically meaningful differences exist between the two scoring systems across different ICU populations.

## Data Availability

The datasets used and/or analyzed during the current study are available from the corresponding author on reasonable request.

## Abbreviations

AI: Artificial intelligence
APACHE: Acute physiology and chronic health evaluation
AUC: Area under the curve
CBC: Complete blood cell
CRP: C-reactive protein
CI: Confidence interval
ECMO: Extracorporeal membrane oxygenation
GCS: Glasgow coma scale
ICU: Intensive care unit
MD: Medical doctor
NPV: Negative predictive value
OR: Odds ratio
ROC: Receiver operating characteristic
SOFA: Sequential organ failure assessment
STROBE: Strengthening the reporting of observational studies in epidemiology
PCT: Procalcitonin
PPV: Positive predictive value
